# Expression of the three components of linear ubiquitin assembly complex in breast cancer

**DOI:** 10.1371/journal.pone.0197183

**Published:** 2018-05-15

**Authors:** Amirhossein Razavirad, Hui Gao, Reza Ghiasvand, Lars-Arne Haldosen, Kazem Zendehdel

**Affiliations:** 1 Cancer Research Center, Cancer Institute of Iran, Tehran University of Medical Sciences, Tehran, Iran; 2 Department of Biosciences and Nutrition, Karolinska Institutet, Huddinge, Stockholm, Sweden; 3 Oslo Centre for Biostatistics and Epidemiology, Institute of Basic Medical Sciences, University of Oslo, Oslo, Norway; University of South Alabama Mitchell Cancer Institute, UNITED STATES

## Abstract

Proteins belonging to the linear ubiquitin assembly complex (LUBAC) are believed to be important in tumorigenesis. LUBAC has been demonstrated to be composed of RBCK1, RNF31 and SHARPIN. The aim of this study was to explore all members of the LUBAC complex as novel biomarkers in breast cancer. We have already reported that RNF31 mRNA levels are higher in breast cancer samples compared to adjacent non-tumor tissue. In this study we extend these findings by demonstrating that the mRNA levels of RBCK1 and SHARPIN are also higher in tumors compared to adjacent non-tumor tissue in the same cross sectional study of samples (*p* < 0.001). In addition, up-regulated mRNA expression of all three members of the LUBAC complex displayed high predictive value in distinguishing tumor tissues from adjacent non-tumor tissue as determined by ROC curve analysis. Furthermore, we investigated whether there is an association between the mRNA and protein expression levels of RBCK1, RNF31 and SHARPIN and clinicopathological parameters including estrogen receptor (ER), progesterone receptor (PR) and human epidermal growth factor receptor (HER2) status and found that RNF31 protein is significantly higher in ERalpha-negative tumors than ERalpha-positive tumors (*p* = 0.034). Collectively, our findings indicate that up-regulated mRNA expression of RNF31, RBCK1 and SHARPIN could potentially be diagnostic biomarkers of breast cancer and RNF31 might be a drug target for ERalpha-negative breast cancers.

## Background

Breast cancer is a heterogeneous disease that consists of several subtypes with different patterns of gene expression, clinical features, treatment regimens and outcomes [1]. Nearly two-thirds of all tumors are dependent on estrogen for continued growth [2]. In the case of breast cancer, most of the known estrogenic effects are mediated through a direct interaction of estrogen with the DNA-binding transcription factor, estrogen receptor alpha (ERalpha) [3, 4]. Thus, ERalpha is a classical drug target in breast cancer using receptor antagonists such as tamoxifen and aromatase inhibitors such as anastrozole [5, 6]. ERalpha regulates the expression of specific sets of genes via a direct interaction with cis-regulatory elements, estrogen-response elements (EREs), of target genes [7]. Additionally, ERalpha can regulate gene expression via interaction with other transcription factors such as activator protein 1 (AP-1) and specific protein 1 (Sp-1) [8, 9]. Although the treatment of breast cancer has been greatly advanced in the past decades due to the discovery of specific predictive, diagnostic and prognostic biomarkers such as ERalpha and human epidermal growth factor receptor 2 (HER2), about one-third of metastatic ERalpha-positive tumors fail to respond to endocrine therapy [10–13]. Thus, an improvement in the ability to predict the outcome of response to endocrine therapy would facilitate accurate recognition and optimal and cost effective treatment of disease [14].

**Fig 1:**
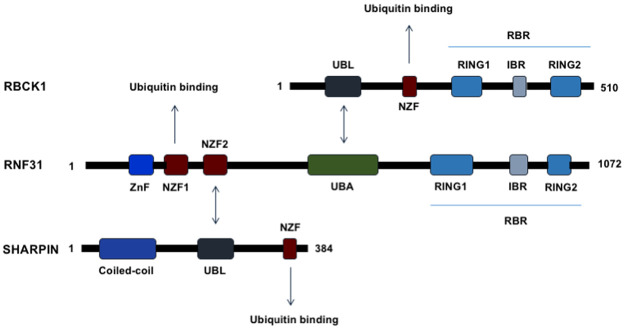
Schematic representation of the members of the LUBAC complex, RBCK1, RNF31 and SHARPIN, including domain structures and active sites. RNF31, the catalytic subunit of the LUBAC complex, interacts with SHARPIN and RBCK1. SHARPIN and RBCK1 bind to the NZF2 and UBA domains of RNF31 via their UBL domains. The RBR domain of RNF31, but not that of RBCK1, plays a key role for the linear ubiquitin chain generating property of LUBAC. Arrows show known interactions between the proteins. Abbreviations: ZnF, zinc finger; NZF, Npl4 zinc finger; UBL, ubiquitin-like domain; IBR, in-between RING domain; RBR, RING-IBR-RING domain.

**Fig 2:**
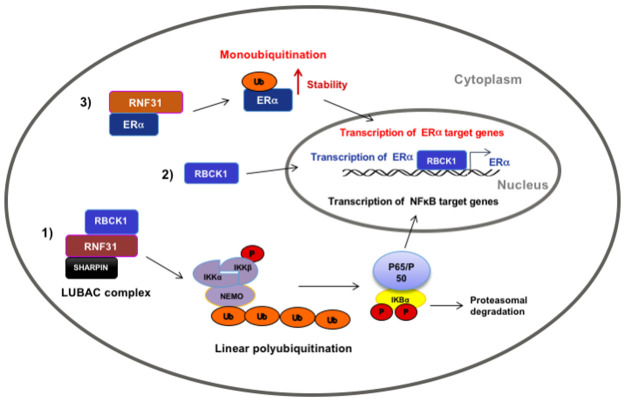
Model showing some pathways that have been suggested to be related to breast cancer. Pathway 1; The LUBAC complex, consisting of RBCK1, RNF31 and SHARPIN, has been shown to activate NF*k*B signaling. Pathway 2; RBCK1 has been shown to regulate ERalpha transcription. Pathway 3; RNF31 has been shown to regulate ERalpha stability presumably via monoubiquitination.

Post-translational modifications play central roles in regulating protein functions and coordinating signaling networks essential for cellular functions. Ubiquitination, involving attachment of the 76-amino-acid protein ubiquitin via its C terminus to an amino group on a target protein, is a well studied and important regulatory modification of proteins [15]. Moreover, it has been shown that defects in the ubiquitination system can cause diseases such as cancer [13]. In addition to form the inter-ubiquitin linkage with the 7 lysine residues of ubiquitin, the amino-terminal methionine of ubiquitin can act as an acceptor site to form the 8^th^ inter-linkage, the so called linear-inter-ubiquitin linkage [16]. Ubiquitination is performed via a cascade of three steps catalyzed by a ubiquitin activating enzyme (E1), a ubiquitin conjugating enzyme (E2) and a ubiquitin ligase enzyme (E3) [17]. The ability of ubiquitin in generating diverse protein-ubiquitin structures leads to different protein fates. [18]. The linear ubiquitin assembly complex (LUBAC) is the only identified E3 ubiquitin ligase complex to date demonstrated to have the capacity to generate linear polyubiquitin chains in cells [19]. LUBAC was identified as a 600-kDa complex. Three related multi-domain proteins are identified to form LUBAC (Figs 1 and 2) [19–21]. They are the Ran Bp-type and C3HC4-type zinc finger-containing protein 1 (RBCK1), the RING finger protein 31 (RNF31), and the SHANK-associated RH domain interacting protein (SHARPIN) [19–21]. RBCK1 and RNF31 are predicted to have E3 ubiquitin ligase function, but SHARPIN does not appear to have any enzymatic activity [21–23]. However, the protein structure of SHARPIN includes a highly conserved ubiquitination superfamily domain, suggesting that it is important in protein ubiquitination [24].

Interestingly, the protein expression levels of the three LUBAC components are independently of each other in different cell types. It is therefore possible that these proteins may also serve functions that are independent of LUBAC activity [16]. Our research group has previously shown that RBCK1 promotes proliferation in ERalpha expressing MCF-7 and T-47D breast cancer cells and suggested that this is due to up-regulation of ERalpha gene and protein expression and down-stream target genes (Fig 2) [25]. In addition, we have recently reported that RNF31 stabilizes ERalpha via a monoubiquitination mechanism, and facilitates ERalpha-dependent proliferation in breast cancer cell lines (Fig 2) [26]. Furthermore, an important role for SHARPIN in tumorigenesis has been demonstrated [27]. The relation between the above functions of RBCK1, RNF31 and SHARPIN and the LUBAC complex, if any, remains to be established. We have recently reported that RNF31 mRNA expression is significantly higher in breast cancer samples compared to adjacent tissue in an Iranian cross sectional study [26]. In this study we extend this observation by determining mRNA and protein expression levels of the two additional components of the LUBAC complex, RBCK1 and SHARPIN as well as RNF31 protein expression in the same cross sectional study of cancer tumors and adjacent non-tumor tissues. Furthermore, we investigate whether there is a diagnostic or predictive value of these genes as well as their correlation with ER, progesterone receptor (PR), HER2 statuses, lymph node involvement, stage and grade.

## Materials and methods

### Tissue collection and tumor specimens

The details of this cross-sectional study have been previously described [28]. Tissue samples of 72 primary breast cancer specimens (range 24–85 years) and 37 adjacent non-tumor tissues were available. For 36 cases, paired samples from tumor and adjacent non-tumor tissues were available. Histologically all tumors were classified as invasive ductal and lobular carcinomas. ER, PR and HER2 statuses were available in 70, 62 and 68 cases and were positive in 47, 35 and 14 cases, respectively (Table 1). Fifty-two of the primary breast tumors were lymph node positive and 20 were lymph node negative. Thirty-eight patients were premenopausal and 32 postmenopausal, and for two patients the menopausal status was not available. The pathological staging was done as recommended by the American Joint Committee on Cancer (AJCC) TNM system. Eight tumors were classified as stage I, 37 as stage II, 25 as stage III and 2 as stage IV. Moreover, 25 patients classified as grade 1, 40 as grade 2, 6 as grade 3 and one as missing a grade. All samples have been provided from the National Tumor Bank of the Cancer Institute of Iran. Written informed consent was obtained from all patients who donated samples to the tumor bank.

**Table 1:** Clinicopathological data of patients.

**ER Status**	
Positive (%)	47 (65.3)
Negative (%)	18 (25.0)
Weak (%)	5 (6.9)
Unknown (%)	2 (2.8)
**PR Status**	
Positive (%)	35 (48.6)
Negative (%)	25 (34.7)
Weak (%)	2 (2.8)
Unknown (%)	10 (13.9)
**HER2 Status**	
Positive (%)	14 (19.4)
Negative (%)	54 (75.0)
Weak (%)	2 (2.8)
Unknown (%)	2 (2.8)

### Ethics statement

The National Research Ethics Committee of I.R of Iran and the Regional Research Ethics committee of Karolinska Institute approved the study (approval numbers: 1-K90.P52 and 2012/774-31/2, respectively).

### Real-time PCR analysis

RNA was extracted from fresh frozen tissues using RNeasy plus Universal Mini Kits (QIAGEN) as per the manufacturer’s instructions. The integrity and concentration of the RNA was assessed using the Agilent Bioanalyzer. Complementary DNA (cDNA) was synthesized using Superscript III First-Strand Synthesis SuperMix (Invitrogen), as per the manufacturer’s instructions. One μg RNA from each sample was used as starting material for cDNA synthesis.

Real-time PCR was run in triplicate in a 7500 ABI real-time PCR thermocycler (Applied Biosystems). ERalpha (ESR1), RBCK1, RNF31 and SHARPIN mRNA expression were determined by TaqMan assay (Hs00174860_ml), TaqMan assay (Hs00246291_ml), TaqMan assay (Hs00215938_m1), TaqMan assay (Hs00229642_ml), respectively. The ubiquitin C (UBC) TaqMan assay (Hs00824723_m1) was used for normalization. The final volume per well was 15 μL. The thermal cycling conditions were 95 °C for 20 seconds once, then repetitively 95 °C for 3 seconds and 60 °C for 30 seconds for all assays.

The mRNA expression was calculated using the ΔCt method by subtracting the average of triplicates of selected genes from the average Ct-value of triplicates of the housekeeping gene UBC as an internal control [29].

### Western blot analysis

Frozen tumor tissue was minced and cells were homogenized in RIPA buffer (Sigma) containing complete mini protease inhibitor cocktail (Roche). Protein extracts were prepared as described previously [30]. Forty μg of protein extract was analyzed by Western blot using RNF31 polyclonal antibody (ab85294) (1:2000), RBCK1 polyclonal antibody (ab38540) (1:400) and SHARPIN polyclonal antibody (ab125188) (1:2000). All antibodies were purchased from Abcam Company.

### Statistical analysis

Student’s t-test was performed to compare continuous variables between two different categorical clinicopathological characteristics including ERalpha, PR, HER2, lymph node status, staging and menopausal status. In addition, one-way ANOVA was used when comparing a continuous variable with several categorical explanatory variables such as BSR grading system. Pearson correlation coefficient was calculated pairwise for all assayed mRNAs in breast tumors and ERalpha-positive tumors. Logistic regression models were fitted with the mRNA expression of the selected genes as the independent variable. Curve estimation regression was also fitted with the mRNA or protein expression levels of interest. Additionally, Chi-square test used to show significant differences between protein expression levels and clinicopathological parameters. ImageJ software was used to quantify the protein expressions and also median was used to stratify the protein expression into two subgroups, low and high.

The ROC (Receiving Operating Characteristic) curve test was used and the area under the curve (AUC) was calculated to summarize and display the distinction between tumor and adjacent non-tumor tissues, as the area under the curve defined previously [31]. An arbitrary level of 5% for statistical significance (two-sided) was considered in all analyses. Statistical analysis was calculated by means of Excel software and SPSS statistical software version 23.

## Results

### mRNA expression levels of RBCK1 and SHARPIN are higher in tumors compared to adjacent tissues

**Fig 3:**
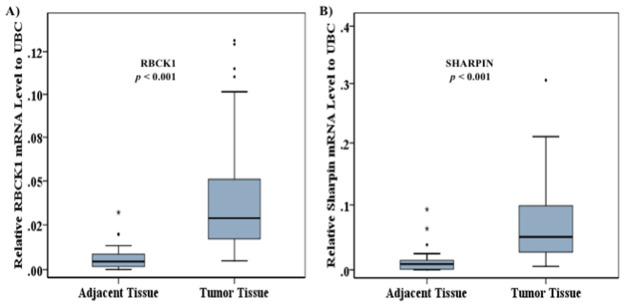
Expression of genes constituting the linear ubiquitin assembly complex comparing tumor and adjacent tissues. The expression levels of RBCK1 (A) and SHARPIN (B) were significantly higher in tumors compared with adjacent tissues (*p* < 0.001). A non-paired model was applied. Gene expression (y-axis) was quantified by real-time PCR and normalized to UBC.

As shown in Fig 3, RBCK1 and SHARPIN mRNA expression levels were significantly higher in 72 tumors compared with 37 adjacent tissues (*p* < 0.001). We have recently reported that RNF31 mRNA expression levels are higher in the same set of tumors compared to adjacent tissues [26]. Thus, all three members of the LUBAC complex are higher in tumors compared to adjacent tissues. Restricting the analysis to samples from the 36 individuals for which paired samples were available gave similar results, including for RNF31 (S1 Fig).

**Fig 4:**
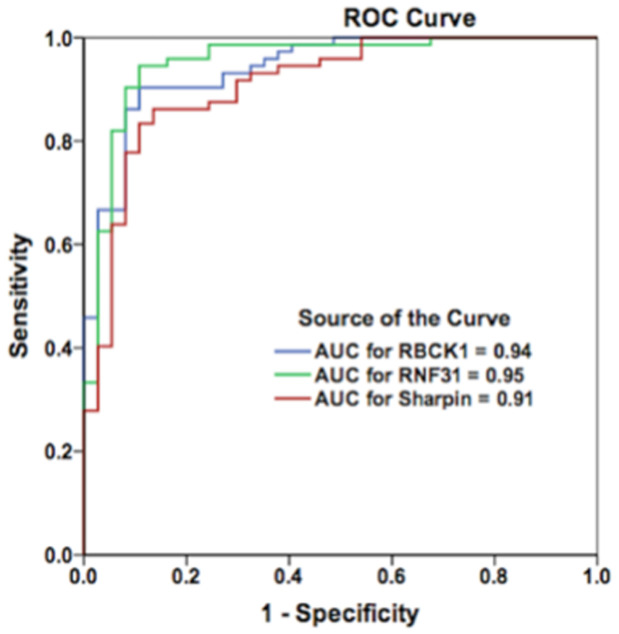
ROC Curve analyses comparing tumor and adjacent tissues. Up-regulated mRNA expression of RNF31, RBCK1 and SHARPIN exhibited high predictive distinction abilities to distinguish tumor tissues from adjacent non-tumor tissues with Area Under the Curve (AUC) equal to 0.95, 0.94 and 0.91, respectively.

ROC Curve analysis revealed that up-regulated RNF31, RBCK1 and SHARPIN mRNA expression had high predictive abilities to distinguish tumor tissues from adjacent non-tumor tissues, with the Area Under the Curves (AUCs) being equal to 0.95, 0.94 and 0.91, respectively (Fig 4).

### mRNA expression levels of LUBAC complex members are strongly correlated to each other in adjacent non-tumor tissues but not in tumor tissues

**Fig 5:**
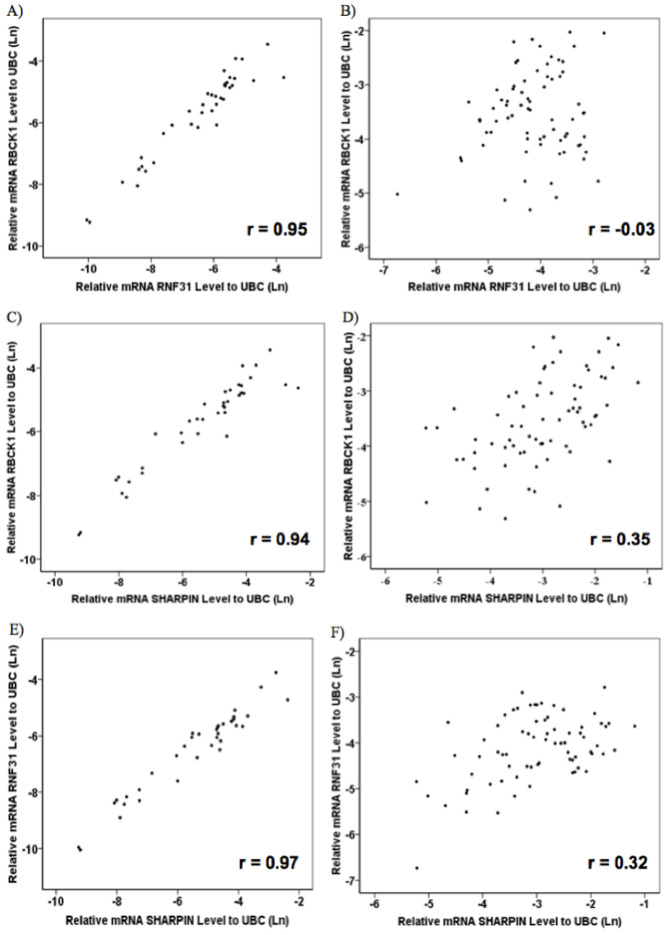
Genes involved in LUBAC complex show strong correlations to each other in adjacent non-tumor tissues. Correlations among the expression of genes involved in the LUBAC complex revealed good pair wise correlations among non-tumor tissues (r > 0.9) (A, C and E), while the correlation diminished or disappeared among tumors (B, D and F). Gene expression was quantified by real-time PCR and normalized to UBC.

We correlated pairwise the mRNA expression levels of LUBAC complex members in 37 adjacent non-tumor tissues and 72 tumor samples. A strong pairwise correlation was observed for mRNA expression levels of LUBAC complex members, in adjacent non-tumor tissues (Fig 5A, 5C and 5E; r > 0.9). These correlations decreased for tumor tissues (Fig 5B, 5D and 5F), as SHARPIN mRNA expression level was moderately correlated with RBCK1 and RNF31 mRNA expression levels (r = 0.52 and r = 0.48, respectively).

### Analysis of the mRNA expression levels of LUBAC complex against ERalpha mRNA expression

As shown in Table 2, there were fair correlations between ERalpha and RBCK1 mRNA expression levels (r = 0.27, *p* = 0.020), and between ERalpha and RNF31 mRNA expression levels (r = 0.25, *p* = 0.036). Restricting the analyses to only ERalpha-positive tumors did not affect the correlation between ERalpha and RNF31 mRNA expression levels (r = 0.29, *p* = 0.042), while ERalpha mRNA expression levels correlated moderately with RBCK1 and SHARPIN mRNA expression levels (r = 0.51, *p* < 0.001 and r = 0.61, *p* < 0.001, respectively). Correlations between SHARPIN and RBCK1 mRNA expression levels (r = 0.57, *p* < 0.001), and between SHARPIN and RNF31 mRNA expression levels (r = 0.56, *p* < 0.001) were not affected by ERalpha status.

**Table 2:** Correlation of ERalpha, RBCK1, RNF31 and SHARPIN mRNA expression levels in breast tumors. Pearson correlation was performed pairwise for all assayed mRNAs in breast tumors and the subgroup of ERalpha-positive tumors.

Paired	Variables	72 tumors		47 ERalpha-positive tumors	
		Pearson correlation	*P* value	Pearson correlation	*P* value
**ERalpha**	**RBCK1**	0.27	0.020*	0.51	< 0.001**
**ERalpha**	**RNF31**	0.25	0.036*	0.29	0.042*
**ERalpha**	**SHARPIN**	0.18	0.136	0.61	< 0.001**
**RBCK1**	**RNF31**	0.15	0.202	0.16	0.270
**RBCK1**	**SHARPIN**	0.52	< 0.001**	0.57	< 0.001**
**RNF31**	**SHARPIN**	0.48	< 0.001**	0.56	< 0.001**

*Statistically significant (*p* < 0.05 in two-tailed test).

**Statistically significant (*p* < 0.01 in two-tailed test).

### Analysis of RBCK1, RNF31 and SHARPIN mRNA expression levels with clinical characteristics

Since clinicopathological data for tumor samples including ERalpha, PR and HER2 protein expression and also clinical staging, histological grading, lymph node and menopausal statuses were available, we further examined the mRNA expression of RBCK1, RNF31 and SHARPIN in relation to these data. We observed a significant increased expression of RBCK1 in tumors having HER2-positive statues compared to HER2-negative tumors [odds ratio (OR) = 2.71, 95% confidence interval (CI): 1.10–6.71)] (Table 3). In addition, we observed that tumors corresponding to stages III and IV exhibited 49% reduced expression of SHARPIN compared to tumors corresponding to stages I and II (OR = 0.51, 95% CI: 0.29–0.90) (Table 4). We also found significant associations between RBCK1 and SHARPIN expression levels and lymph node status, where tumors categorized as having more than three lymph node involvement displayed more than 50% reduced expression levels of RBCK1 and SHARPIN compared with the two other groups (OR = 0.36, 95% CI: 0.16–0.85 and OR = 0.49, 95% CI: 0.24–0.98, respectively) (Table 4). Moreover, we observed reduced expression of RBCK1 (OR = 0.54, 95% CI: 0.28–0.98), RNF31 (OR = 0.36, 95% CI: 0.16–0.80) and SHARPIN (OR = 0.56, 95% CI: 0.32–0.97) in tumors from postmenopausal women compared with premenopausal women (Table 4). We found no association between the mRNA expression of RBCK1, RNF31 and SHARPIN with histological grading.

**Table 3:** ORs and 95% CIs for the mRNA expression of investigated genes in tumors with clinicopathological parameters. ORs and CIs for RBCK1, RNF31 and SHARPIN from the logistic regression model, stratified on some parameters including ER, PR and HER2 statuses.

Parameter	OR (95% CI)		
	RBCK1	RNF31	SHARPIN
**ER status**			
** Negative**	1	1	1
** Positive**	0.96 (0.49–1.86)	1.02 (0.51–2.03)	0.55 (0.30–1.01)
** *P* value**	0.896	0.962	0.055
**PR status**			
** Negative**	1	1	1
** Positive**	1.04 (0.56–1.95)	0.76 (0.39–1.48)	0.73 (0.43–1.22)
** *P* value**	0.89	0.426	0.226
**HER2 Status**			
** Negative**	1	1	1
** Positive**	2.71 (1.10–6.71)	0.73 (0.32–1.64)	1.90 (0.91–3.98)
** *P* value**	**0.030** *	0.441	0.09

**Abbreviations**: OR: odds ratio, CI: confidence interval.

*Statistically significant (*p* < 0.05 in two-tailed test).

**Table 4:** ORs and 95% CIs for the mRNA expression of investigated genes in tumors with clinicopathological parameters. ORs and CIs for RBCK1, RNF31and SHARPIN from the logistic regression model, stratified on some parameters including menopausal and lymph node statuses, stage and grade.

Parameter	OR (95% CI)		
	RBCK1	RNF31	SHARPIN
**Menopausal Status**			
** Pre-menopause**	1	1	1
** Post-menopause**	0.54 (0.28–1.03)	0.36 (0.16–0.80)	0.56 (0.32–0.97)
** *P* value**	0.055	**0.012** *	**0.040** *
**Lymph Node Status**			
** 0**	1	1	1
** 1–3**	0.82(0.37–1.80)	0.86(0.38–1.93)	0.88(0.44–1.76)
** 3<**	0.36(0.16–0.85)	1.26(0.54–2.93)	0.49(0.24–0.98)
** *P* value for trend**	**0.026** *	0.625	0.065
**Stage**			
** I- II**	1	1	1
** III- IV**	1.5 (0.80–2.82)	1.40(0.70–2.82)	0.51(0.29–0.90)
** *P* value**	0.211	0.342	**0.020** *
**Grade**			
** 1**	1	1	1
** 2**	0.91 (0.48–1.75)	1.66 (0.80–3.47)	1.56 (0.90–2.74)
** 3**	1.02 (0.32–3.27)	0.70 (0.23–2.18)	1.32 (0.50–3.50)
***P* value for trend**	0.954	0.202	0.274

**Abbreviations**: OR: odds ratio, CI: confidence interval.

*Statistically significant (*p* < 0.05 in two-tailed test).

Finally, since age could constitute a potential confounder, we analyzed the possible correlation of the mRNA expression levels of RBCK1, RNF31 and SHARPIN with age, but no correlation was observed (data not shown).

### RNF31 protein is highly expressed in ERα-negative tumors

**Fig 6:**
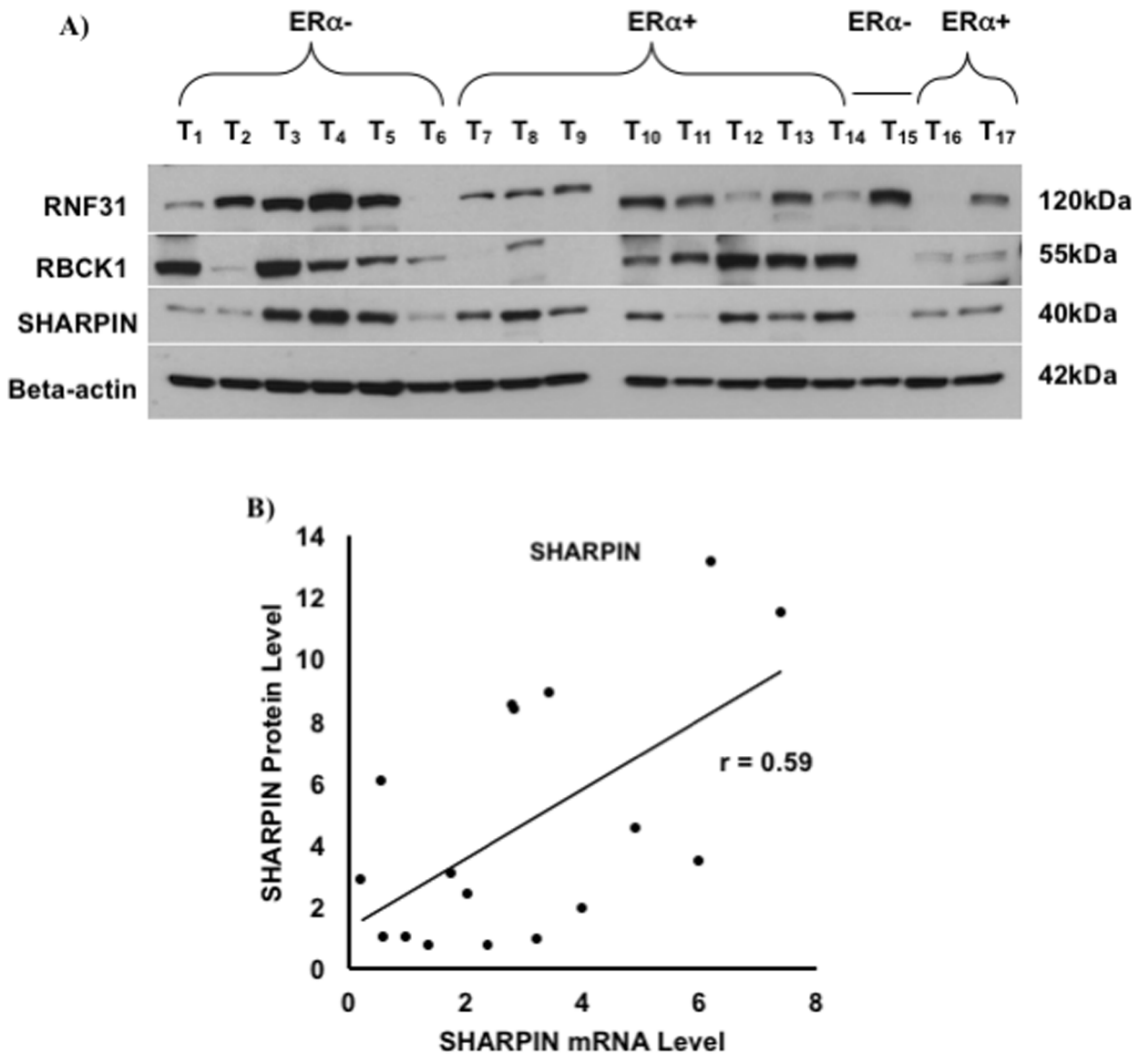
Western blot analysis of RNF31, RBCK1 and SHARPIN for breast tumors. A) Equal amounts of protein (40 μg) from 17 tumor tissues were loaded. Beta-actin was used as loading control. RNF31, RBCK1 and SHARPIN displayed bands with apparent molecular weights of approximately 120, 55 and 40 kDa, respectively. B) ImageJ software was used to quantify the Western blot analysis results of protein expression of interest. The protein expression levels were normalized to beta-actin as a loading control. Curve estimation regression was fitted with the protein expression levels of interest and their corresponding mRNA expression levels. Among three members of LUBAC complex only SHARPIN protein expression levels were correlated moderately with its corresponding mRNA levels (r = 0.59).

We performed analysis of protein levels for 17 breast tumor specimens including 10 ERalpha-positive and 7 ERalpha-negative tumors. Samples were selected based on differential mRNA expression of RNF31, RBCK1 and SHARPIN, respectively (Fig 6A and S2–S5 Figs). We found that RNF31 protein was highly expressed in most ERalpha-negative tumors relative to 10 ERalpha-positive tumor specimens (*p* = 0.034, Table 5). In addition, the results showed that SHARPIN protein expression levels were correlated moderately with its corresponding mRNA levels (r = 0.59, Fig 6B). However, RNF31 and RBCK1 protein expression levels did not correlate with the expression levels of their corresponding mRNAs (data not shown). Additionally, analyzing the protein expression of three LUBAC components against some clinicopathological parameters including PR, lymph node, stage and grade statuses did not show significant differences (Table 5).

**Table 5:** The correlation of RBCK1, RNF31 and SHARPIN protein expression levels with clinicopathological parameters. ImageJ software was used to quantify the western blot analysis results of protein expression of interest. The protein expression levels were normalized to beta-actin as a loading control. Median was used to classify the protein expression levels into two subgroups, as low and high. Chi-square test used to show significant differences between protein expression levels and some clinicopathological parameters including ER, PR, lymph node, stage and grade statuses.

Parameters	Protein Expression^¶^ (No. = 17)								
	RBCK1		*P* value	RNF31		*P* value	SHARPIN		*P* value
	Low	High		Low	High		Low	High	
**ERalpha Status**									
** Negative**	3	4	0.772	2	5	**0.034** *	4	3	0.772
** Positive**	5	5		8	2		5	5	
**PR Status**									
** Positive**	5	5	0.772	5	5	0.377	5	5	0.772
** Negative**	3	4		5	2		4	3	
**Lymph node No**.									
** 0**	1	2	0.240	1	2	0.529	1	2	0.425
** <3**	5	2		5	2		5	3	
** >3**	2	5		4	3		3	4	
**Stage**									
** 2**	6	4	0.201	6	4	0.906	6	4	0.486
** 3**	2	5		4	3		3	4	
**Grade**									
** 2**	2	5	0.201	5	2	0.377	3	4	0.486
** 3**	6	4		5	5		6	4	

^**¶**^ Median values of protein expressions, quantified by ImageJ, and normalized to beta-actin

*Statistically significant (*p* < 0.05 in two-tailed test).

## Discussion

This study was undertaken to examine mRNA expression levels of components of the LUBAC complex in breast cancer tissue compared to adjacent tissue as well as their correlation to clinical characteristics and molecular markers of breast cancer. Also, the protein expression levels of LUBAC complex components in breast tumors were examined and analyzed against clinicopathological characteristics.

LUBAC complex is known as a 600-kDa protein complex consisting of three proteins, RNF31, RBCK1 and SHARPIN. In addition, LUBAC has been shown to function as a regulator of the NF-*k*B pathway with activated NF-*k*B stimulating proliferation and blocking programmed cell death (apoptosis) in human breast cancer [32]. However, as earlier mentioned the protein expression levels of the three LUBAC components are independently expressed in different cell types. Therefore, it is more likely that these proteins play functions that are independent of LUBAC complex activity [16].

RNF31 was originally cloned from breast cancer cells based on its higher mRNA expression compared to normal breast cell lines [33]. In line with this, we have also recently reported that RNF31 is significantly overexpressed in breast cancer tumors compared to adjacent non-tumor tissues and can facilitate ERalpha-dependent proliferation in breast cancer cell lines through monoubiquitination of ERalpha protein [34]. However, our new finding of the elevated RNF31 protein expression in ERα-negative tumors could also indicate an ERalpha-independent role of RNF31 in breast cancer.

We have previously reported that RBCK1 mRNA expression correlates positively with ERα mRNA expression in a limited set of ERα-positive breast cancer samples [25]. Our results reported in the present study of breast cancer samples from the Iranian population are consistent with the previous study, as we found a significant positive correlation between RBCK1 mRNA expression and ERalpha mRNA expression levels (r = 0.51, *p* < 0.001). Furthermore, our results indicating an inverse association between RBCK1 mRNA expression and lymph node involvement are in agreement with a previous study, suggesting a less aggressive tumor phenotype when RBCK1 is highly expressed [35].

Our results for SHARPIN demonstrated a significantly elevated mRNA with high discrimination ability (AUC = 0.91) to differentiate tumors from adjacent non-tumor tissues are consistent with recently published study, where De Melo et al systematically analyzed the mRNA expression of SHARPIN using Oncomine datasets derived from 17 studies [36]. They found that SHARPIN mRNA expression levels are significantly elevated in invasive ductal breast carcinomas compared to non-tumor breast tissues and that the expression of this gene has an ability to discriminate breast tumors from non-tumor tissues (AUC = 0.83) [36]. However, our results for SHARPIN do not support any association between SHARPIN gene expression and breast cancer progression, where SHARPIN expression significantly decrease in high stages.

In addition, our analysis of the pairwise correlation of three LUBAC genes revealed the high expression with low correlations for tumors against the low expression with perfect correlations (r > 0.9) for adjacent non-tumor tissues. This finding could be due to the accumulation of genomic mutations and tumor cellular environments, leading to up-regulation of genes in different types of cancer [37, 38].

We compared RNF31, RBCK1 and SHARPIN mRNA and protein expression in a subset of breast tumors. SHARPIN protein expression levels were correlated moderately with its corresponding mRNA levels whilst no correlation was observed for RNF31 and RBCK1 protein levels with their corresponding mRNA levels. A recent study indicated that cellular concentrations of proteins often correlate moderately with the abundances of their corresponding mRNAs [39]. This study showed that the squared Pearson correlation coefficient (R^2^) can be as low as ~0.4 depending on the system, indicating that only about 40% of the variation in protein concentrations can be derived from the corresponding mRNA concentrations. This modest correlation between mRNA and protein levels could reflect regulation of translation and protein degradation [39]. In addition, breast cancer is a heterogeneous disease that the heterogeneity can occur within tumors (intra-tumor heterogeneity) and/or between tumors (inter-tumor heterogeneity). Thus, tumor heterogeneity can impose significant challenges in detecting the subtypes of breast cancer and strategies of effective therapies [40].

While ERalpha has already been well documented to play an important role in the etiology and progression of breast cancer, RBCK1, RNF31 and SHARPIN has recently emerged as potential new players in tumorigenesis. Follow-up of patients and evaluation of the expression of RBCK1, RNF31 and SHARPIN with tumor recurrence and overall survival of patients may further shed lights on the prognostic role of this complex on breast cancer.

## Supporting information

S1 Fig Expression of genes involved in linear ubiquitin assembly complex comparing tumor and adjacent tissues. The expression levels of RBCK1 (A), RNF31 (B) and SHARPIN (C) were significantly higher in tumors compared with adjacent tissues for paired samples (*p* < 0.001). Gene expression (y-axis) was quantified by real-time PCR and normalized to UBC.
(TIFF)

S2 Fig Western blot analysis for 17 clinical samples with RNF31 antibody. Equal amounts of protein (40 μg) from 17 tumor tissues were loaded. RNF31 showed two bands with apparent molecular weights of approximately 230 and 120 kDa, respectively. RNF31 depletion for MCF7 cells confirmed the accurate band with molecular weight of 120 kDa (data not shown).
(TIFF)

S3 Fig Western blot analysis for 17 clinical samples with RBCK1 antibody. Equal amounts of protein (40 μg) from 17 tumor tissues were loaded. RBCK1 displayed bands with different molecular weights. Positive control for RBCK1 detected the accurate band with molecular weight of 55 kDa.
(TIFF)

S4 Fig Western blot analysis for 17 clinical samples with SHARPIN antibody. Equal amounts of protein (40 μg) from 17 tumor tissues were loaded. SHARPIN displayed the specific band with apparent molecular weight of 40 kDa.
(TIFF)

S5 Fig Western blot analysis for 17 clinical samples with beta-actin antibody. Beta-actin was used as a loading control. Beta-actin detected the specific band with apparent molecular weight of 42 kDa.
(TIFF)

## Abbreviations

AUC: Area under the curve
cDNA: complementary deoxyribonucleic acid
CI: Confidence interval
ER: Estrogen receptor
HER2: Human epithermal growth factor receptor 2
LUBAC: linear ubiquitin assembly complex
mRNA: messenger ribonucleic acid
NF*k*B: Nuclear factor kappa B
OR: Odds ratio
PR: Progesterone receptor
RBCK1: Ran Bp-type and C3HC4-type zinc finger-containing protein 1
RNF31: RING finger protein 31
ROC: Receiving operating characteristic
RT-qPCR: Reverse transcriptase quantitative polymerase chain reaction
SBR grading system: Scarff, Bloom, Richardson, grading system
SHARPIN: SHANK-associated RH domain interacting protein
UBC: Ubiquitin C
