# Supplementary figures and images for: Expression of the three components of linear ubiquitin assembly complex in breast cancer
Source: PLoS One. 2018 May 15;13(5):e0197183. doi: 10.1371/journal.pone.0197183 (PMC5953448; doi:10.1371/journal.pone.0197183)

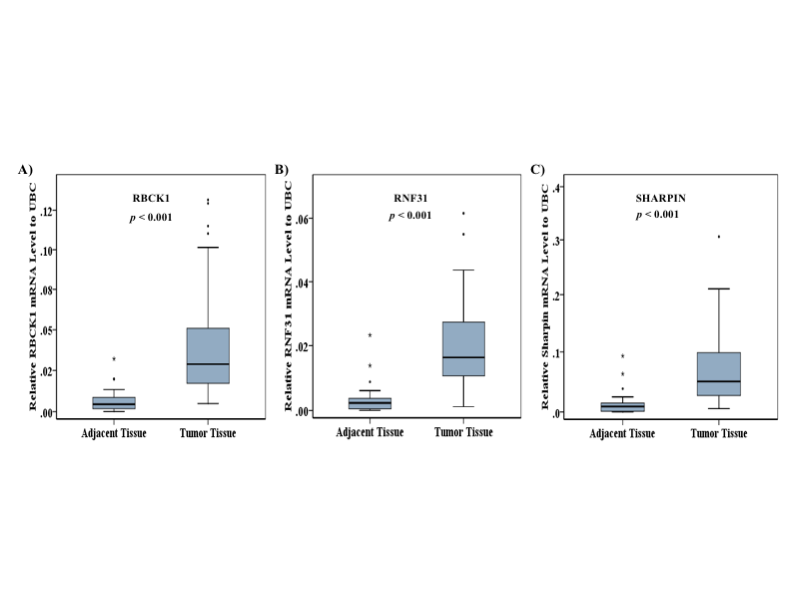

Supplement: S1 Fig — The expression levels of RBCK1 (A), RNF31 (B) and SHARPIN (C) were significantly higher in tumors compared with adjacent tissues for paired samples (p < 0.001). Gene expression (y-axis) was quantified by real-time PCR and normalized to UBC. (TIFF) [file pone.0197183.s001.tiff]

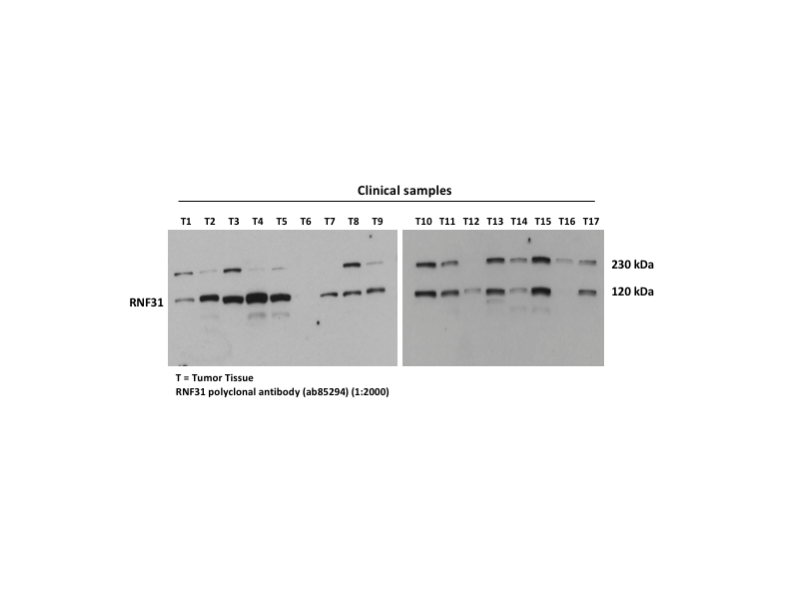

Supplement: S2 Fig — Equal amounts of protein (40 μg) from 17 tumor tissues were loaded. RNF31 showed two bands with apparent molecular weights of approximately 230 and 120 kDa, respectively. RNF31 depletion for MCF7 cells confirmed the accurate band with molecular weight of 120 kDa (data not shown). (TIFF) [file pone.0197183.s002.tiff]

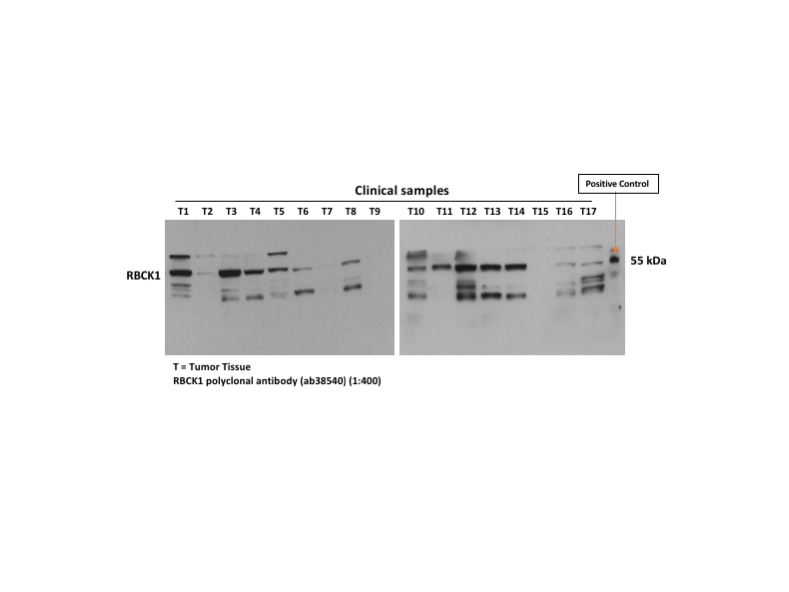

Supplement: S3 Fig — Equal amounts of protein (40 μg) from 17 tumor tissues were loaded. RBCK1 displayed bands with different molecular weights. Positive control for RBCK1 detected the accurate band with molecular weight of 55 kDa. (TIFF) [file pone.0197183.s003.tiff]

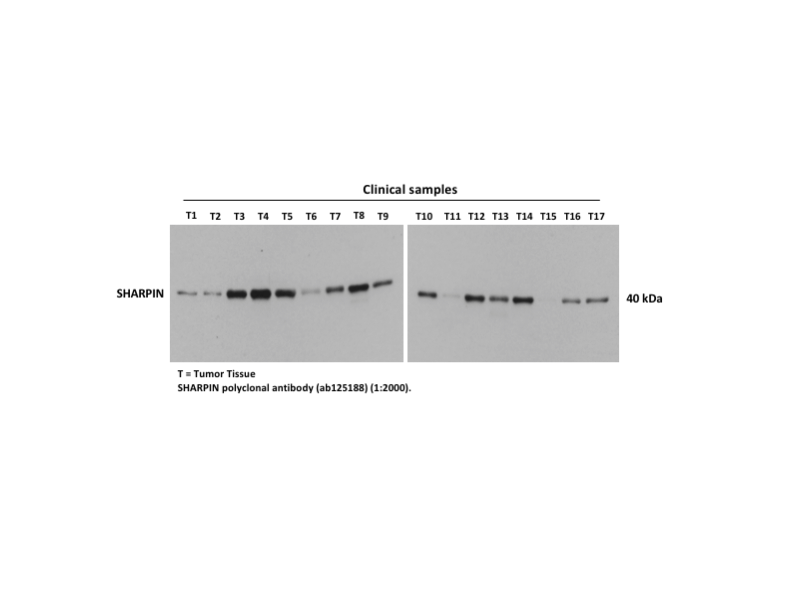

Supplement: S4 Fig — Equal amounts of protein (40 μg) from 17 tumor tissues were loaded. SHARPIN displayed the specific band with apparent molecular weight of 40 kDa. (TIFF) [file pone.0197183.s004.tiff]

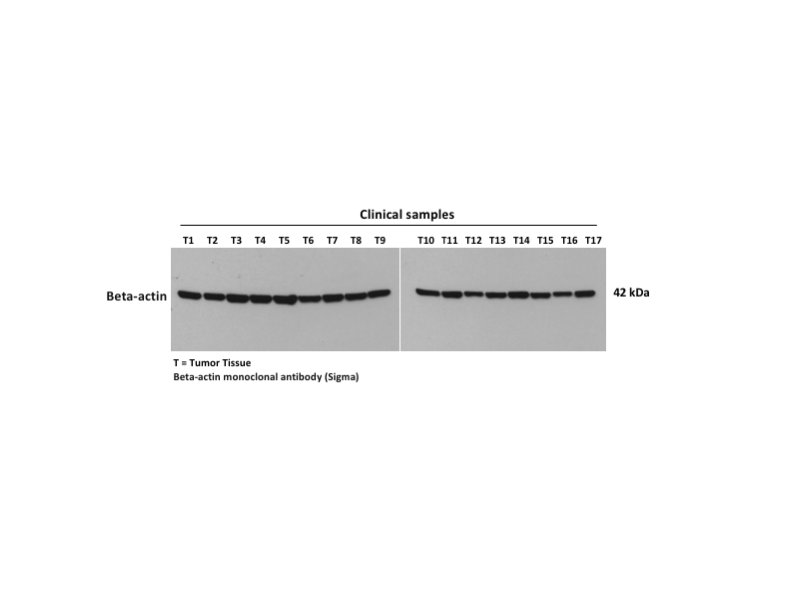

Supplement: S5 Fig — Beta-actin was used as a loading control. Beta-actin detected the specific band with apparent molecular weight of 42 kDa. (TIFF) [file pone.0197183.s005.tiff]
